# Transcription factor FoxM1 is the downstream target of c-Myc and contributes to the development of prostate cancer

**DOI:** 10.1186/s12957-018-1352-3

**Published:** 2018-03-20

**Authors:** Huafeng Pan, Yudi Zhu, Wei Wei, Siliang Shao, Xin Rui

**Affiliations:** Department of Urology, Ningbo No.2 Hospital, No.41 Xibei Street, Ningbo, 315010 Zhejiang Province People’s Republic of China

**Keywords:** Prostate cancer, FoxM1, c-Myc, Promoter

## Abstract

**Background:**

Prostate cancer is a common malignancy and the second leading cause of cancer death in men. Elevated expression of the transcription factor FoxM1 and c-Myc has been identified in prostate cancer. However, the potential mechanism of elevated FoxM1 and c-Myc to the development of prostate cancer has not been identified.

**Methods:**

In this report, the mRNA level of FoxM1 and c-Myc was detected in 30 prostate cancer and para-cancer tissues. Then, we detected the expression level of FoxM1 by real-time PCR and Western blot after disturbance of the expression level of c-Myc in PC-3 cells. Whether c-Myc could bind to FoxM1 promoter was identified by ChIP assay. Finally, the migratory, invasive, and proliferative abilities in FoxM1 overexpressing and silencing PC-3 cells were detected by wound healing, transwell assay, CCK-8 assays, and Ki-67 protein level.

**Results:**

We found that the expression level of FoxM1 and c-Myc were both increased in prostate cancer samples compared with para-cancer samples. The expression level of FoxM1 was changed consistent with the protein level of c-Myc. ChIP assay detected the direct binding of c-Myc in FoxM1 gene promoter. Lastly, overexpression of FoxM1 increased the migratory, invasive, and proliferative abilities of PC-3 cells, and its downregulation significantly decreased the migratory, invasive, and proliferative abilities.

**Conclusions:**

In conclusion, FoxM1 was significantly increased in prostate cancer samples, and it could regulate the proliferative and invasive ability of prostate cancer cells which might be a new target for prostate cancer. Besides, c-Myc could regulate the expression level of FoxM1 by directly binding to its gene promoter.

## Background

Prostate cancer is one of the common malignant tumors in western countries [1]. At present, the incidence of prostate cancer ranks the second highest of all malignancies in the world and the mortality rate ranks fifth [2], although the incidence and mortality of prostate cancer in China is much lower than other developed countries. In recent years, the number of persons who diagnosed with prostate cancer in China has obviously increased with the aging of the population and the change of people’s eating habits. [3]. So, studying the molecular mechanism of prostate cancer is important to its identification and treatment.

The dysregulation of tumor suppressor genes and oncogenes in cancer cells is the most common event in the pathogenesis of prostate cancer [4]. These changes could be the result and at the same time be the cause of tumor. Studying the function of those genes is still important to clarify the mechanism of the pathogenesis of tumor. FoxM1 is an important member of the FOX gene family, which is only specifically expressed in the proliferative stage cells [5]. It can also promote the transition from cell cycle G1 phase to S phase and G2 phase to M phase by inducing the expression of cell cycle-related factors [6, 7]. So, it is suggested that FoxM1 gene could regulate the cell proliferation ability. FoxM1 could also regulate cell differentiation and apoptosis [8–10]. Recent studies have confirmed that abnormal expression of FoxM1 is highly associated with the generation and progression of various human malignancies including breast cancer, liver cancer, lung cancer, and bladder cancer [11–14]. Its expression level is often associated with the degree of malignancy which indicated its potential to be used as a new target for clinical cancer therapy. However, the expression level of FoxM1 in prostate cancer and its clinical significance in the progression of prostate cancer have not been fully studied.

c-Myc, a member of the MYC gene family, is involved in regulating a lot of biological activities [15]. From now on, c-Myc has been found overexpressed in colon cancer, breast cancer, lung cancer, and prostate cancer [16–19]. It is a bidirectional regulatory gene in promoting cell proliferation and inducing cell apoptosis. c-Myc could promote proliferative ability of cells in the stimulation of hematopoietic growth factor. In the absence of hematopoietic growth factor, c-Myc could mediate apoptosis. c-Myc gene also belongs to the apoptosis regulatory gene [20]. In addition, c-Myc could also regulate cell cycle and cell metabolism [21]. In the quiescent cells, the expression level of c-Myc is very low. It accumulates as the initial response gene and maintains at high level throughout the cell cycle when stimulated with growth factors. Once the expression level of c-Myc is out of control, it can lead to tumorigenesis. c-Myc regulates its target genes by directly binding or recruiting histone modification enzyme to the gene’s promoter region [22, 23]. The predicted c-Myc binding sites in FoxM1’s promoter suggested its regulatory role for FoxM1 expression.

In this study, the increased mRNA level of FoxM1 and c-Myc in prostate cancer tissues was further confirmed. Then, we mostly focused on the mechanism of the increased FoxM1 expression level to the pathogenesis of prostate cancer and the regulatory factors of FoxM1. According to ChIP assay and real-time PCR assay, we proved FoxM1 was regulated by c-Myc. Further experiments showed that FoxM1 could promote the proliferative and invasive ability of prostate cancer cells. Altogether, the increased expression of FoxM1 in prostate tissues might be caused by c-Myc overexpression, and FoxM1 promoted the development of prostate cancer.

## Methods

### Sample collection

All tissues were obtained from patients who underwent surgical procedures for prostate cancer treatment or diagnoses in Ningbo No.2 Hospital. In total, 30 prostate cancer tissues and 30 para-cancer tissues were obtained for experiments in this study. All the tissues were collected for RNA isolation.

### Cell culture

Human PC-3 cells were purchased from American Type Culture Collection. All the cells were cultured in 1640 media supplemented with 10% fetal bovine serum in a 5% CO_2_ humidified atmosphere at 37 °C.

### RNA isolation and real-time PCR

After PC-3 cells were transfected with c-Myc overexpression plasmid and small interference RNAs (the small interference RNA of c-Myc was synthesized by RiboBio), the cells were collected by centrifugation and discarded the supernatant. According to the operation instructions, the RNA was extracted by Trizol reagent. After extraction, the RNA was quantified and purified by ultraviolet spectrophotometer. The integrity of RNA was identified by 0.8% agarose gel electrophoresis. Then, 1 μg of total RNA was reversed by ABI reverse transcriptional kit according to the manufacturer’s instruction. Reverse transcription reaction procedure was as follows: 25 °C for 10 min, 37 °C for 120 min, and 85 °C for 5 min. Lastly, the real-time PCR amplification scheme was carried out according to the manufacturer’s instructions of ROCH. The primers used to detect the expression of FoxM1 and c-Myc were as follows: FoxM1 forward: GGAGCAGCGACAGGTTAAGG, reverse: GTTGATGGCGAATTGTATCATGG c-Myc forward: GGCTCCTGGCAAAAGGTCA; reverse: CTGCGTAGTTGTGCTGATGT.

### Western blot

After PC-3 cells were transfected with c-Myc and FoxM1 overexpression plasmid and small interference RNAs, cells were collected by centrifugation to discard the supernatant. The cells are collected by centrifugation and discarded. The cell deposition was re-suspended in cell lysis buffer (50 mM Tris, pH 7.4, 0.5% NP-40 and 0.01% SDS) containing protease inhibitors. After 5 min of boiling in SDS-loading buffer, the supernatant was separated by sodium dodecyl sulfate (SDS)-polyacrylamide gel. Then, the protein in the gel was transferred to the PVDF membrane. In order to prevent the non-specific protein and protein interactions, the PVDF membrane was blocked at room temperature in Tris saline Tween (TST) buffer containing 5% non-fat milk. The antibodies were diluted in TST buffer (c-Myc, 1:4000 (Santa Cruz,); FoxM1, 1:2000 (Santa Cruz,); β-Actin, 1:5000 (Santa Cruz)); PVDF membranes were maintained TST buffer containing diluted antibody and shaked at 4 °C overnight. The PVDF membrane was washed with TST buffer the next day, and then, a secondary antibody with peroxidase conjugation was incubated at room temperature for 1 h. Finally, ECL solution was prepared in a dark room, and exposure time was measured according to fluorescence intensity.

### Cell viability

PC-3 cells were grown into 96-well cell culture plates for 5 × 10^5^ cells each well. After cells were transfected with c-Myc and FoxM1 overexpression plasmid and small interference RNAs, PC-3 cells were harvested in medium 24 and 48 h after normal culture for proliferation detection. Cell proliferation ability was detected according to the manufacturer’s instruction of CCK8 assay kit. Absorbance was detected using a microplate reader at the wavelength of 450 nm.

### Wound healing assay

PC-3 cells were transfected with FoxM1 overexpression plasmid and small interference RNA on the second day of the plate. After 6 h transfection, the sterile 10-μl white tip was used to draw a straight line from top to bottom at the bottom of a 3.5-cm dish and replaced with serum-free medium. At the same time, pictures were taken as 0 h in × 40 microscope. Following culture for 24 and 48 h, pictures were taken as 24 h in × 40 microscope.

### Transwell assay

The invasive ability of PC-3 cells was detected by using Matrigel transmembrane invasion assay. The transwell chambers were coated with Matrigel. PC-3 cells were transfected either with FoxM1 overexpressing plasmid or with FoxM1 siRNA on the second day of the plate. After transfection for 24 h, PC-3 cells were harvested for calculating and plating into the upper chamber and bottom wells were filled with complete medium. After incubation for 24 h, scraping with a cotton swab was used to remove cells from the upper surface of the filter. The invaded cells were stained with crystal violet solution after being fixed with methanol. Lastly, the number of penetrated cells of five randomly selected fields were counted, then the mean number was calculated.

### ChIP assay

ChIP assays were conducted according to the manufacturer (Upstate) of ChIP assay kit. For quantitative ChIP analysis, real-time PCR was carried out by applying SYBR Green PCR Master Mix according to the instructions of the Takara manufacturer. Then, 1:4, 1:16, 1:16, and 1:25 diluted input samples were used to make the relative quantitative standard curves. All PCR results from non-precipitate samples were transferred into their respective input standard curve to normalize the number of cells and primers efficiency. The procedure of real-time PCR is applied according to the method described in the report. Δ Ct value in the first uses the following formula: Δ Ct = Ct (sample) − Ct (input), Δ Δ Ct (Δ Ct sample − Δ Ct negative control), and fold difference (2 (− Δ Δ Ct treatment)/2 (− Δ Δ Ct control) was derived. Primers used in ChIP assay are as follows: F1: ATCCAGCCTTTGCACGCCTGA; R1: AGAGGTTAATGATACTTTGAGT; F2: TGGAGACGGTGTCTCCCTCTGT; R2: AGCGAAACCGCTTCTCTACTA.

### Statistical analysis

All the results in this report were showed as the mean value ± standard deviation (SD). Student’s *t* test was used for all the statistical analyses. Each experiment in this research was repeated at least in triplicate. Each *p* value less than 0.05 is thought to be with great significance.

## Results

### The expression level of FoxM1 and c-Myc was increased in prostate cancer tissues

**Fig. 1:**
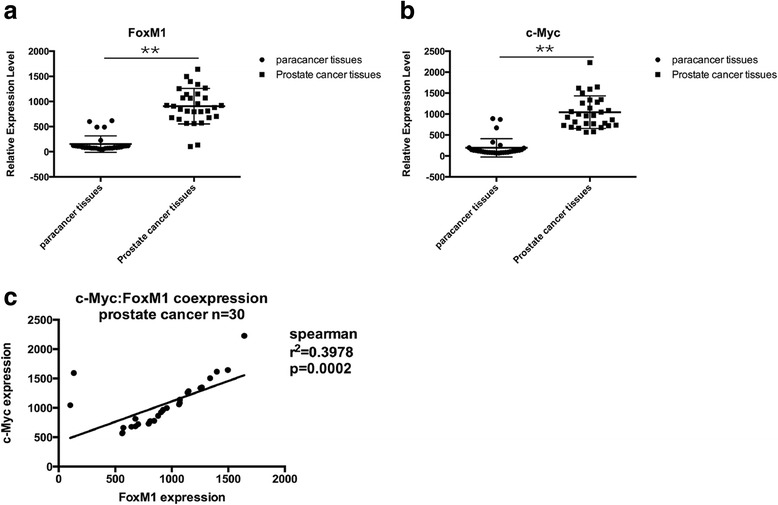
The expression level of FoxM1 and c-Myc in prostate cancer tissues and para-cancer tissues. Thirty prostate cancer samples and para-cancer samples were collected and isolated for obtaining RNA. **a** The expression level of FoxM1 was detected by real-time PCR. **b** The expression level of c-Myc was detected by real-time PCR. **c** The correlation analysis of the expression level of FoxM1 and the expression level of c-Myc. (**p < 0.01)

Thirty prostate cancer samples and para-cancer samples were collected and isolated for obtaining RNA. The relative expression level of FoxM1 and c-Myc was detected by real-time PCR assay. Compared with normal para-cancer tissues, the relative mRNA levels of FoxM1 (Fig. 1a) and c-Myc (Fig. 1b) were both increased in prostate cancer tissues. The increase of FoxM1 and c-Myc expression was seen in 87.2% prostate cancer samples. In addition, we also analyzed the correlation between the mRNA level of FoxM1 and c-Myc. The expression level of FoxM1 was highly correlated with the expression level of c-Myc (Fig. 1c).

### FoxM1 was regulated by c-Myc

**Fig. 2:**
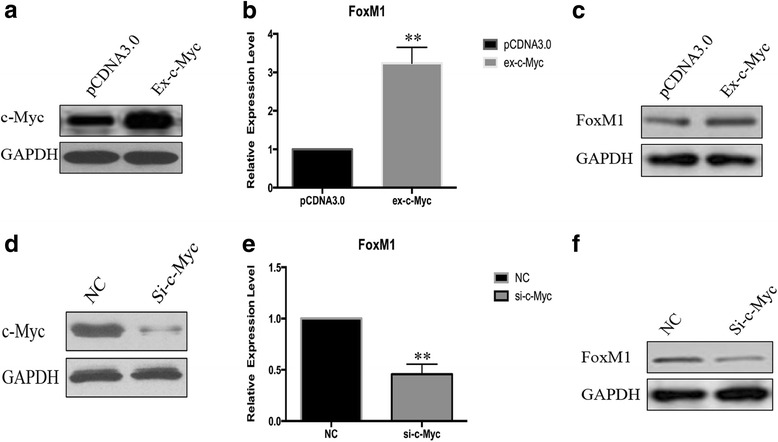
c-Myc was a regulator of FoxM1 in prostate cancer cells. PC-3 cells were transfected with c-Myc overexpression plasmid and small interference RNA. **a** The c-Myc overexpression effect was evaluated by Western blot. **b** The expression level of FoxM1 was detected by real-time PCR in c-Myc overexpression group and control group. **c** The protein level of FoxM1 was evaluated by Western blot. **d** The interference effect was evaluated by Western blot. **e** The expression level of FoxM1 was detected by real-time PCR in c-Myc interference group and control group. **f** The protein level of FoxM1 was evaluated by Western blot. (**p < 0.01)

To clarify the mechanism of the increasing expression level of FoxM1 in prostate cancer tissues, we tried to find the regulatory transcription factors of FoxM1. According to the transcription factor prediction in FoxM1 gene’s promoter, we focused on an important oncogene c-Myc which also increased in prostate cancer tissues. So, we constructed c-Myc overexpression plasmid and confirmed the overexpression efficiency by Western blot. As shown in Fig. 2b, c, both the protein level and mRNA level of FoxM1 were significantly increased in c-Myc overexpressed PC-3 cells. In addition, we knocked down the expression level of c-Myc by interference RNA, then we detected the expression level of FoxM1 was consistent with the change of c-Myc as shown in Fig. 2e, f. Altogether, FoxM1 was regulated by transcription factor c-Myc.

### c-Myc bound to FoxM1 gene promoter

**Fig. 3:**
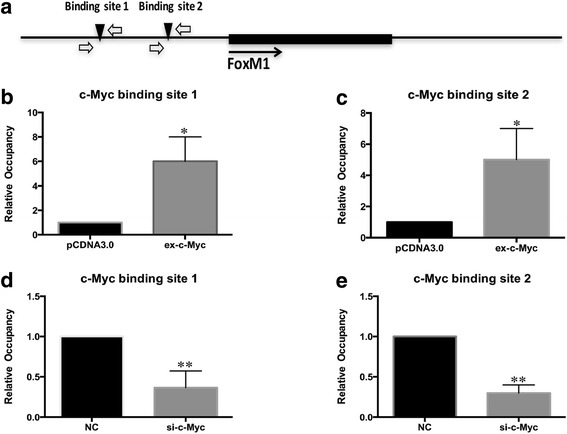
c-Myc bound to FoxM1 gene promoter. **a** The schematic graph of relative positions of c-Myc binding sites in FoxM1 gene promoter. **b** PC-3 cells were transfected with c-Myc overexpression plasmid and small interference RNA. The binding of c-Myc on site 1 of FoxM1 promoter in c-Myc overexpression group and control group was detected by ChIP assay. **c** The binding of c-Myc on site 2 of FoxM1 promoter in c-Myc overexpression group and control group was detected by ChIP assay. **d** The binding of c-Myc on site 1 of FoxM1 promoter in c-Myc interference group and control group was detected by ChIP assay. **e** The binding of c-Myc on site 2 of FoxM1 promoter in c-Myc interference group and control group was detected by ChIP assay. (*p < 0.05, **p < 0.01)

In order to further confirm whether c-Myc could bind to the promoter region of FoxM1, we applied ChIP assay to detect the binding of c-Myc on FoxM1’s promoter in the condition of changing c-Myc expression level in PC-3 cells. Quantitative ChIP technology demonstrated increased c-Myc binding on the predicted c-Myc binding sites after overexpressing c-Myc in PC-3 cells (Fig. 3b, c). Similarly, the binding of c-Myc was decreased in c-Myc silencing PC-3 cells (Fig. 3d, e).

### FoxM1 regulated the proliferative ability of PC-3 cells

**Fig. 4:**
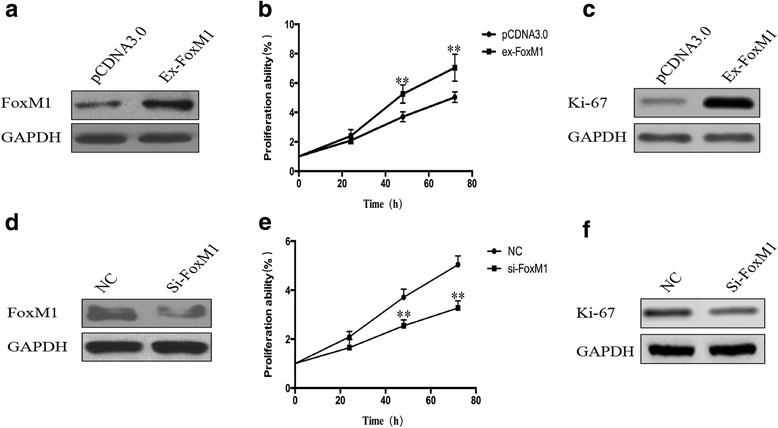
FoxM1 regulated the proliferative ability of PC-3 cells. PC-3 cells were transfected with FoxM1 overexpression plasmid and small interference RNA. **a** The FoxM1 overexpression effect was evaluated by Western blot. **b** The CCK-8 assay result showed that the PC-3 cells’ proliferative ability was increased in FoxM1 overexpression group compared with control. **c** Ki-67 protein level was increased in FoxM1 overexpression cells by Western blot. **d** The interference effect was evaluated by Western blot. **e** The CCK-8 assay result showed that the PC-3 cells’ proliferative ability was decreased in FoxM1 knock down group compared with control. **f** Ki-67 protein level was decreased in FoxM1 silencing cells. (**p < 0.01)

PC-3 cells were transfected with FoxM1 overexpression plasmid and interference RNAs to investigate whether FoxM1 could regulate the proliferative ability of prostate cancer cells. The overexpression and interference efficiency were evaluated by Western blot (Fig. 4a, d). The proliferative ability of PC-3 cells transfected with FoxM1 overexpression plasmid and control plasmid was evaluated by CCK8 assay, and the protein level of Ki-67 and the proliferative ability of PC-3 cells were increased by FoxM1 (Fig. 4b, c). The proliferative ability of PC-3 cells transfected with FoxM1 silencing RNAs was significantly decreased (Fig. 4e, f). These results demonstrated that FoxM1 could regulate the proliferative ability of prostate cancer cells.

### FoxM1 regulated the invasive and migratory ability of PC-3 cells

**Fig. 5:**
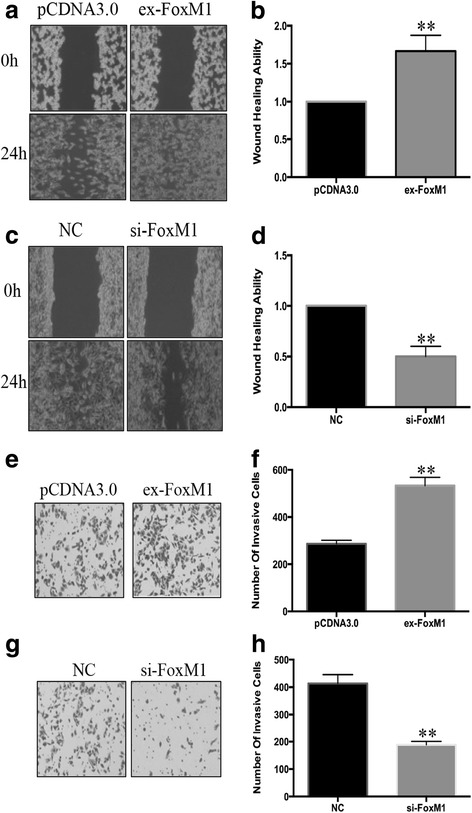
FoxM1 regulated the invasive and migratory ability of PC-3 cells. PC-3 cells were transfected with FoxM1 overexpression plasmid and small interference RNA. **a** The migratory ability of PC-3 cells transfected with FoxM1 overexpression plasmid was evaluated by wound healing assay. **b** Graphical representation of **a**. **c** The migratory ability of PC-3 cells transfected with FoxM1 small interference RNA was evaluated by wound healing assay. **d** Graphical representation of **c**. **e** The invasive ability of PC-3 cells transfected with FoxM1 overexpression plasmid was evaluated by transwell assay. **f** The statistical analysis of invasive ability of PC-3 cells transfected with FoxM1 overexpression plasmid. **g** The invasive ability of PC-3 cells transfected with FoxM1 small interference RNA was evaluated by transwell assay. **h** The statistical analysis of invasive ability of PC-3 cells transfected with FoxM1 small interference RNA. (**p < 0.01)

Next, we tried to identify whether FoxM1 could regulate the prostate cancer cells’ invasive and migratory ability. After transfected PC-3 cells with FoxM1 overexpression plasmid and interference RNAs, the invasive and migratory ability of PC-3 cells were evaluated by wound healing and transwell assay. Results showed that the invasive and migratory ability of PC-3 cells was increased by FoxM1 (Fig. 5b, f). On the contrary, the invasive and migratory ability of PC-3 cells was significantly decreased in FoxM1 silencing cells (Fig. 5d, h). These results suggested that FoxM1 could regulate the invasive and migratory ability of prostate cancer cells, then promote the development of prostate cancer.

## Discussion

Abnormal proliferative and invasive abilities are the phenotypes of cancer cells; we found the increased proliferative and invasive abilities in prostate cancers were possibly caused by FoxM1. In this report, we found increased mRNA level of FoxM1 was caused by c-Myc which was a new mechanism in prostate cancer.

We found the expression level of FoxM1 was increased in prostate cancer tissues which was consistent with the reports of the other cancers. FoxM1 was an important regulator to the proliferative and invasive ability of prostate cancer cells which contributed to the development of prostate cancer. As an important transcription factor, FoxM1 could regulate a wide range of other biological processes, including differentiation, metabolism, and apoptosis. In the study of FoxM1 in gastric cancer, FoxM1 could inhibit cell apoptosis and promote proliferation of gastric cells [24]. FoxM1 also played a vital role in promoting angiogenesis and lymph node metastasis by regulating the expression of MMP in gastric cancer [25]. Besides, the researchers found FoxM1 could participate in regulating the telomerase activity in gastric cancer cells [26]. In hepatocellular carcinoma, FoxM1 could regulate cell cycle arrest, and the expression of cyclin B1, p27, and cyclin D1 was all changed in FoxM1 knock out MHCC-97H cells [27]. So, studying the other functions of FoxM1 in prostate cancer was important to fully understand its role in the progression of prostate cancer.

c-Myc could regulate the expression level of FoxM1 by binding to its promoter region which was a newfound mechanism in prostate cancer. Besides, its regulatory mechanism includes the indirect regulatory mechanism mediated by other proteins [28]. For example, c-Myc could regulate its target genes by interacting with transformation/transcription domain-associated protein (TRRAP) and other histone acetyltransferase [29]. The specific mechanism of c-Myc in regulating FoxM1 by directly binding or being assisted by other proteins still needs further investigation. Except transcription activation, the other studies found that c-Myc possesses transcriptional inhibition activity [28]. It depends on the sequence nearby c-Myc binding sites. Both the increased expression of FoxM1 and c-Myc would promote the development of prostate cancer, and they might contribute to this disease by different mechanisms. In this work, c-Myc upregulated the expression of FoxM1 to promote the cancer development which might be used as a target in clinical treatment.

The regulatory mechanism of FoxM1 was complex; c-Myc was one of the important factors to the increased expression level of FoxM1 in PC-3 cells. FoxM1 was regulated by a wide variety of transcription factors, enzyme, and microRNAs. From the previous report, TNF-α could induce the upregulation of FoxM1 in hepatocellular cancers [30]. In breast cancer, FoxM1 was the downstream target of HER2, then contributes to the pathogenesis of breast cancer [31]. In cervical cancer, E6 oncoprotein augmented the expression of FoxM1 through MZF1/NKX2 [32]. All these FoxM1 regulatory mechanisms in prostate cancer need further investigation.

## Conclusion

In conclusion, we found increased expression of FoxM1 in prostate cancer samples compared with normal prostate samples. FoxM1 contributed to the pathogenesis of prostate cancer by regulating prostate cancer cells’ proliferative, invasive, and migratory abilities. It might be used as a novel target for clinical treatment of prostate cancer. Besides, we found a new regulatory mechanism of FoxM1 in prostate cancer that was c-Myc could regulate the expression of FoxM1 by directly binding to its promoter.

## References

[1] Roshandel G, Boreiri M, Sadjadi A, Malekzadeh R (2014). A diversity of cancer incidence and mortality in West Asian populations. Ann Glob Health.

[2] Duong Q, Hill CL, Janitz AE, Campbell JE (2016). Trends in lung and bronchus, prostate, female breast, and colon and rectum cancers incidence and mortality in Oklahoma and the United States from 1999 to 2012. J Okla State Med Assoc.

[3] Zhao R, Cheng G, Wang B, Qin C, Liu Y, Pan Y, Wang J, Hua L, Zhu W, Wang Z (2017). BMI and serum lipid parameters predict increasing risk and aggressive prostate cancer in Chinese people. Oncotarget.

[4] Turner DP, Watson DK (2008). ETS transcription factors: oncogenes and tumor suppressor genes as therapeutic targets for prostate cancer. Expert Rev Anticancer Ther.

[5] Wang X, Quail E, Hung NJ, Tan Y, Ye H, Costa RH (2001). Increased levels of forkhead box M1B transcription factor in transgenic mouse hepatocytes prevent age-related proliferation defects in regenerating liver. Proc Natl Acad Sci U S A.

[6] Wei Y, Sun Q, Zhao L, Wu J, Chen X, Wang Y, Zang W, Zhao G (2016). LncRNA UCA1-miR-507-FOXM1 axis is involved in cell proliferation, invasion and G0/G1 cell cycle arrest in melanoma. Med Oncol.

[7] Kelleher FC, O'Sullivan H (2016). FOXM1 in sarcoma: role in cell cycle, pluripotency genes and stem cell pathways. Oncotarget.

[8] Chen Y, Liu Y, Ni H, Ding C, Zhang X, Zhang Z (2017). FoxM1 overexpression promotes cell proliferation and migration and inhibits apoptosis in hypopharyngeal squamous cell carcinoma resulting in poor clinical prognosis. Int J Oncol.

[9] Wen N, Wang Y, Wen L, Zhao SH, Ai ZH, Wang Y, Wu B, Lu HX, Yang H, Liu WC, Li Y (2014). Overexpression of FOXM1 predicts poor prognosis and promotes cancer cell proliferation, migration and invasion in epithelial ovarian cancer. J Transl Med.

[10] Ustiyan V, Wert SE, Ikegami M, Wang IC, Kalin TV, Whitsett JA, Kalinichenko VV (2012). Foxm1 transcription factor is critical for proliferation and differentiation of Clara cells during development of conducting airways. Dev Biol.

[11] Yu CP, Yu S, Shi L, Wang S, Li ZX, Wang YH, Sun CJ, Liang J (2017). FoxM1 promotes epithelial-mesenchymal transition of hepatocellular carcinoma by targeting Snai1. Mol Med Rep.

[12] Inoguchi S, Seki N, Chiyomaru T, Ishihara T, Matsushita R, Mataki H, Itesako T, Tatarano S, Yoshino H, Goto Y, Nishikawa R, Nakagawa M, Enokida H (2014). Tumour-suppressive microRNA-24-1 inhibits cancer cell proliferation through targeting FOXM1 in bladder cancer. FEBS Lett.

[13] Wei P, Zhang N, Wang Y, Li D, Wang L, Sun X, Shen C, Yang Y, Zhou X, Du X (2015). FOXM1 promotes lung adenocarcinoma invasion and metastasis by upregulating SNAIL. Int J Biol Sci.

[14] Abdeljaoued S, Bettaieb I, Nasri M, Adouni O, Goucha A, El Amine O, Boussen H, Rahal K, Gamoudi A (2017). Overexpression of FOXM1 is a potential prognostic marker in male breast cancer. Oncol Res Treat.

[15] Aprelikova O, Chen K, El Touny LH, Brignatz-Guittard C, Han J, Qiu T, Yang HH, Lee MP, Zhu M, Green JE (2016). The epigenetic modifier JMJD6 is amplified in mammary tumors and cooperates with c-Myc to enhance cellular transformation, tumor progression, and metastasis. Clin Epigenetics.

[16] Kugimiya N, Nishimoto A, Hosoyama T, Ueno K, Enoki T, Li TS, Hamano K (2015). The c-MYC-ABCB5 axis plays a pivotal role in 5-fluorouracil resistance in human colon cancer cells. J Cell Mol Med.

[17] Wang J, Jia Y, Zhao S, Zhang X, Wang X, Han X, Wang Y, Ma M, Shi J, Liu L. BIN1 reverses PD-L1-mediated immune escape by inactivating the c-MYC and EGFR/MAPK signaling pathways in non-small cell lung cancer. Oncogene. 2017; 10.1038/onc.2017.217 28714960

[18] Sadeghi S, Hojati Z, Tabatabaeian H (2017). Cooverexpression of EpCAM and c-Myc genes in malignant breast tumours. J Genet.

[19] Li E, Liu L, Li F, Luo L, Zhao S, Wang J, Kang R, Luo J and Zhao Z. PSCA promotes prostate cancer proliferation and cell-cycle progression by up-regulating c-Myc. Prostate. 2017;77(16):1563–1572. 10.1002/pros.23432 28971496

[20] Shortt J, Johnstone RW. Oncogenes in cell survival and cell death. Cold Spring Harb Perspect Biol. 2012;4 10.1101/cshperspect.a009829 PMC3504432 23209150

[21] Polioudakis D, Bhinge AA, Killion PJ, Lee BK, Abell NS, Iyer VR (2013). A Myc-microRNA network promotes exit from quiescence by suppressing the interferon response and cell-cycle arrest genes. Nucleic Acids Res.

[22] Chen L, Iraci N, Gherardi S, Gamble LD, Wood KM, Perini G, Lunec J, Tweddle DA (2010). p53 is a direct transcriptional target of MYCN in neuroblastoma. Cancer Res.

[23] Tikhanovich I, Zhao J, Bridges B, Kumer S, Roberts B, Weinman SA (2017). Arginine methylation regulates c-Myc-dependent transcription by altering promoter recruitment of the acetyltransferase p300. J Biol Chem.

[24] Okada K, Fujiwara Y, Takahashi T, Nakamura Y, Takiguchi S, Nakajima K, Miyata H, Yamasaki M, Kurokawa Y, Mori M, Doki Y (2013). Overexpression of forkhead box M1 transcription factor (FOXM1) is a potential prognostic marker and enhances chemoresistance for docetaxel in gastric cancer. Ann Surg Oncol.

[25] Yu J, Wang X, Li Y, Tang B (2017). Tanshinone IIA suppresses gastric cancer cell proliferation and migration by downregulation of FOXM1. Oncol Rep.

[26] Zeng J, Wang L, Li Q, Li W, Bjorkholm M, Jia J, Xu D (2009). FoxM1 is up-regulated in gastric cancer and its inhibition leads to cellular senescence, partially dependent on p27 kip1. J Pathol.

[27] Wu QF, Liu C, Tai MH, Liu D, Lei L, Wang RT, Tian M, Lu Y (2010). Knockdown of FoxM1 by siRNA interference decreases cell proliferation, induces cell cycle arrest and inhibits cell invasion in MHCC-97H cells in vitro. Acta Pharmacol Sin.

[28] Dang CV (1999). c-Myc target genes involved in cell growth, apoptosis, and metabolism. Mol Cell Biol.

[29] Liu X, Tesfai J, Evrard YA, Dent SY, Martinez E (2003). c-Myc transformation domain recruits the human STAGA complex and requires TRRAP and GCN5 acetylase activity for transcription activation. J Biol Chem.

[30] Xia L, Mo P, Huang W, Zhang L, Wang Y, Zhu H, Tian D, Liu J, Chen Z, Zhang Y, Chen Z, Hu H, Fan D, Nie Y, Wu K (2012). The TNF-alpha/ROS/HIF-1-induced upregulation of FoxMI expression promotes HCC proliferation and resistance to apoptosis. Carcinogenesis.

[31] Francis RE, Myatt SS, Krol J, Hartman J, Peck B, McGovern UB, Wang J, Guest SK, Filipovic A, Gojis O, Palmieri C, Peston D, Shousha S, Yu Q, Sicinski P, Coombes RC, Lam EW (2009). FoxM1 is a downstream target and marker of HER2 overexpression in breast cancer. Int J Oncol.

[32] Chen PM, Cheng YW, Wang YC, Wu TC, Chen CY, Lee H (2014). Up-regulation of FOXM1 by E6 oncoprotein through the MZF1/NKX2-1 axis is required for human papillomavirus-associated tumorigenesis. Neoplasia.

